# A bio-inspired convolution neural network architecture for automatic breast cancer detection and classification using RNA-Seq gene expression data

**DOI:** 10.1038/s41598-023-41731-z

**Published:** 2023-09-05

**Authors:** Tehnan I. A. Mohamed, Absalom E. Ezugwu, Jean Vincent Fonou-Dombeu, Abiodun M. Ikotun, Mohanad Mohammed

**Affiliations:** 1 School of Mathematics, Statistics, and Computer Science, University of KwaZulu-Natal, King Edward Avenue, Pietermaritzburg Campus, Pietermaritzburg, 3201 KwaZulu-Natal South Africa; 2 Unit for Data Science and Computing, North-West University, Potchefstroom, South Africa

**Keywords:** Computational models, Machine learning, Cancer

## Abstract

Breast cancer is considered one of the significant health challenges and ranks among the most prevalent and dangerous cancer types affecting women globally. Early breast cancer detection and diagnosis are crucial for effective treatment and personalized therapy. Early detection and diagnosis can help patients and physicians discover new treatment options, provide a more suitable quality of life, and ensure increased survival rates. Breast cancer detection using gene expression involves many complexities, such as the issue of dimensionality and the complicatedness of the gene expression data. This paper proposes a bio-inspired CNN model for breast cancer detection using gene expression data downloaded from the cancer genome atlas (TCGA). The data contains 1208 clinical samples of 19,948 genes with 113 normal and 1095 cancerous samples. In the proposed model, Array-Array Intensity Correlation (AAIC) is used at the pre-processing stage for outlier removal, followed by a normalization process to avoid biases in the expression measures. Filtration is used for gene reduction using a threshold value of 0.25. Thereafter the pre-processed gene expression dataset was converted into images which were later converted to grayscale to meet the requirements of the model. The model also uses a hybrid model of CNN architecture with a metaheuristic algorithm, namely the Ebola Optimization Search Algorithm (EOSA), to enhance the detection of breast cancer. The traditional CNN and five hybrid algorithms were compared with the classification result of the proposed model. The competing hybrid algorithms include the Whale Optimization Algorithm (WOA-CNN), the Genetic Algorithm (GA-CNN), the Satin Bowerbird Optimization (SBO-CNN), the Life Choice-Based Optimization (LCBO-CNN), and the Multi-Verse Optimizer (MVO-CNN). The results show that the proposed model determined the classes with high-performance measurements with an accuracy of 98.3%, a precision of 99%, a recall of 99%, an f1-score of 99%, a kappa of 90.3%, a specificity of 92.8%, and a sensitivity of 98.9% for the cancerous class. The results suggest that the proposed method has the potential to be a reliable and precise approach to breast cancer detection, which is crucial for early diagnosis and personalized therapy.

## Introduction

Breast cancer (BRCA) is the most prevalent cancer in women, and it is characterized by the uncontrolled division and expansion of breast cells^1,2^. Industrialized and developing nations are experiencing increased cancer incidence and prevalence^3^. Breast cancer incidence and death rates are serious public health concerns^4^. The World Health Organization (WHO) estimates that in 2023 there will be more than 2.3 million new instances of breast cancer globally and 685,000 deaths from the disease^5^. Early detection and accurate diagnosis of BRCA are crucial for effective treatment and personalized therapy. Morphological characteristics play an important role in detecting and diagnosing breast cancer. When a sample of breast tissue is obtained through a biopsy or surgical procedure, a pathologist examines the tissue under a microscope and looks for specific morphological features that are associated with breast cancer, such as abnormal cell growth, changes in cell shape or size, and the presence of cancerous cells. These morphological characteristics can provide important information about the type, stage, and aggressiveness of the cancer, which can help guide treatment decisions and predict patient outcomes. While morphological examination remains a crucial tool in the detection and diagnosis of breast cancer, it has some limitations^6–9^.

The limitations of morphological characteristics in detecting and diagnosing breast cancer can lead to bias and difficulty in identification by physicians^10^. Advancements in microarray technology and the more recent Next Generation Sequencing (NGS) has made gene expression profiling of patients widely available, resulting in the collection of gene expression datasets corresponding to various diseases. This shift has marked a significant transformation in personalized medicine, departing from traditional descriptive "morphological" classification approaches towards a more comprehensive strategy that considers clinical characteristics and immunohistochemical biomarkers. Today, gene expression profiling has become well-integrated into routine clinical practice^11,12^. Breast cancer researchers have examined gene expression profiling in-depth, and clinical oncologists are starting to use the findings of these studies in their daily practices. Also, the early detection and treatment of different cancer types have benefited from mining gene expression level data^13^. Many methods are designed to accurately predict breast cancer based on gene expression data^14–16^. Computational techniques are becoming increasingly crucial in detecting breast cancer due to the rapid growth of computer technology. However, the use of computational techniques is affected by gene expression dataset characteristics such as small dataset sizes, excessive dimensionality, and unbalanced data^17^. Several machine learning, deep learning, and metaheuristic techniques have been created and applied to detect and classify cancer using gene expression data.

Khalsan et al.^18^ presented an extensive overview of recent cancer research works that utilize gene expression data from various types of cancer, including kidney, breast, ovarian, lung, liver, gallbladder and central nervous system. The review encompasses several facets of machine learning in cancer research, including cancer classification, cancer prediction, identification of biomarker genes, and using microarray and RNA-Seq data. Yuan et al.^19^ applied different methods of machine learning for the detection of lung cancer through the use of gene expression data. A novel computational method for detecting breast cancer was proposed by Wang et al.^20^ based on incorporating random forest (RF), Monte Carlo feature selection (MCFS), rough set-based rule learning, SVM, and dagging. A deep learning method that uses Stacked Denoising Autoencoder (SDAE) to identify genes that can effectively differentiate between tumor and healthy cases of breast cancer was proposed by Danaee et al.^21^. BRCA gene expression data from TCGA and gene expression omnibus (GEO) was analyzed by Jia et al.^22^. They used differentially expressed genes (DEG) and weighted gene co-expression network analysis (WGCNA) to select the most significant genes. A deep learning model combined with an artificial intelligence-based feature selection method (AIFSDL-PCD) using gene expression data was proposed by Alshareef et al.^23^ for detecting prostate cancer.

The field of cancer prediction using machine and deep learning methods based on gene expression data has seen significant progress in recent years. However, despite the progress in predicting cancer using machine and deep learning methods based on gene expression data, the existing models have some issues affecting their performance. These issues include choosing the feature representation, optimal architecture, including the number of layers and nodes, suitable model parameters, and picking the best values for weights and bias are critical steps in improving performance^24–26^. Moreover, selecting the most suitable learning rates and regularization parameters can affect the model's ability to generalize to unseen data. Therefore, this paper aims to resolve these issues by finding a precise prediction model and advancing the state-of-the-art use of CNN to classify gene expression data using metaheuristic methods to optimize the CNN model.

Metaheuristic algorithms are optimization algorithms that search for solutions by exploring a large search space and iteratively improving candidate solutions. They have the ability to handle NP-hard problems, which are computationally intractable problems that cannot be solved using exact methods, by providing near-optimal solutions within a reasonable amount of time^27–29^. Metaheuristic optimization algorithms have been identified as an effective tool for solving large-scale optimization problems in bioinformatics. Many of these problems can be classified as NP-hard; thus, researchers have relied heavily on metaheuristic methods to address them. The metaheuristic methods allow for the efficient solution of large-scale samples while minimizing the use of computational resources. Despite the availability of various optimization methods, metaheuristic optimization algorithms are instrumental in solving optimization problems due to their flexibility in providing high-quality optimization solutions in a relatively short amount of computing time^30^. The use of metaheuristics models assists in solving the problems of high dimensionality, the complexity of variable relationships and noisy data peculiar to gene expression data. In addition, metaheuristics models can handle noisy and non-linear data by incorporating techniques such as randomization and simulated annealing to escape from local optima^31^. Chakraborty et al.^32^ presented a metaheuristic method for skin disease classification based on an artificial neural network. In MotieGhader et al.^33^, metaheuristic methods, including GA, WCC, PSO, CUK, ICA, LA, HTS, ACO, FOA, DSOS, and LCA, with an SVM classifier were used for the detection of breast cancer based on mRNA and micro-RNA expression data.

This paper proposes using the metaheuristic model EOSA-CNN for breast cancer detection using gene expression data^34^. EOSA is a new optimization algorithm with excellent performance track records in different application domains^35–39^. It is population-based and bio-inspired, developed by taking clues from the Ebola virus's effective propagation. The algorithm's framework was designed based on the spread of Ebola disease (EVD)^34,40^. This research makes significant contributions by introducing a bio-inspired CNN model for detecting breast cancer using gene expression data from the TCGA repository. The AAIC method is used for pre-processing to remove the outliers' samples, thereafter, normalization and filtration were used. Furthermore, we converted the pre-processed data into 2D images that can be utilized in the CNN architecture. The study also proposes a hybrid of the proposed CNN architecture that employs the EOSA to enhance the classification performance. The proposed model showed its ability to classify the tumor and normal samples with high accuracy and reliability. In our proposed model, the best combination of weights required for the feature extraction is obtained using the EOSA algorithm to handle the classification problem. Therefore, this study presents a hybrid model that combines the proposed CNN and EOSA for the process of classification based on BRCA gene expression data. Consequently, in this study, the main contributions are as follows:

- Applying various pre-processing techniques (such as removing outliers, normalizing, and filtering) to prepare the gene expression data.
- Transforming the gene expression data into two-dimensional images.
- Proposal of a novel bio-inspired CNN architecture for the detection of breast cancer.
- Introducing a hybrid model that combines the proposed CNN and EOSA for the classification process.
- Assessing and comparing the proposed model with other metaheuristic algorithms combined with the proposed CNN.

The rest of the paper is structured as follows: a detailed account of the related work is given in Section “Related work”, while Section “Model Methodology” describes the model technology discussing the CNN Architecture and the Ebola Optimization Algorithm CNN Model (EOSA-CNN) along with the associated algorithms. Section “Experimentation, results and discussion” presents the experimental results with a discussion of the results. Comparison with results from the literature, the strengths and limitations of the model are also enumerated. Finally, the conclusion and the recommendations for future work are presented in Section “Conclusion and future work”.

## Related work

As earlier noted, several machine learning, deep learning, and metaheuristic techniques have been created and applied to detect and classify cancer using gene expression data. Yuan et al.^19^ applied different machine-learning methods for detecting lung cancer through gene expression data. The Monte Carlo and incremental feature selection methods were used to identify the most important genes. Then, SVM and random forest (RF) were implemented, and their performances were compared. The results indicated that SVM achieved an accuracy, sensitivity, specificity, precision, and F1-measure of 100%, 93.2%, 96.7%, 93.9%, and 96.9%, respectively. These results are higher than those obtained using RF. Wang et al.^20^ proposed a novel computational method called Patient-derived tumor xenograft (PDX) for breast cancer detection by incorporating Monte Carlo feature selection, RF, rough set-based rule learning, SVM, and dagging. In the work of Danaee et al.^21^ proposed, a deep learning approach that uses Stacked Denoising Autoencoder (SDAE) to identify genes that can effectively differentiate between tumor and healthy cases of breast cancer was proposed. They tested the efficacy of the extracted features using an artificial neural network (ANN), SVM, and SVM-RBF. The results showed that using the SDAE method with SVM-RBF achieved the highest accuracy of 98.26%.

Jia et al.^22^ analyzed BRCA gene expression data from TCGA and GEO using differentially expressed genes (DEG) and weighted gene co-expression network analysis (WGCNA) to select the most significant genes. Twenty-three hub genes were then identified using a protein–protein interaction (PPI) network. They applied SVM, decision tree (DT), Bayesian network (BN), ANN, and convolutional neural network (CNN-LeNet and CNN-AlexNet), and the results showed that ANN has the best performance with an average accuracy of 97.36%. Elbashir et al.^41^ developed a lightweight CNN model for detecting breast cancer using RNASeq gene expression data. They first pre-processed the data by removing outliers, normalization and filtration. Then they converted the gene expression profiles into 2-D images. Thereafter, they applied a lightweight CNN model for the classification. From their result, their model achieved an accuracy of 98.76. Alshareef et al.^22^ proposed a deep learning model with an artificial intelligence-based feature selection method for prostate cancer detection (AIFSDL-PCD) using gene expression data. In addition, a feature selection (FS) method based on a chaotic invasive weed optimization (CIWO) to select the optimal genes revealed the novelty of their approach. Their results showed sensitivity, specificity, precision, F1-measure, and accuracy of 97.25%, 97.25%, 0.967%, 97.14%, 97.28%, and 97.19%, respectively. Chakraborty et al.^32^ presented a metaheuristic method for skin disease classification based on an artificial neural network. Their proposed method, a non-dominated sorting genetic algorithm—II (NNNSGAII), was used to train an ANN. The proposed method obtained 87.92% accuracy, 94.2% precision, 87.5% recall, and 90.73% F-measure.

MotieGhader et al.^33^ used metaheuristic methods, including world competitive contest (WCC), league championship algorithm( LCA), GA, particle swarm optimization (PSO), ant colony optimization (ACO), imperialist competitive algorithm (ICA), learning automata (LA), heat transfer optimization algorithm (HTS), Forest optimization algorithm (FOA), discrete symbiotic organisms search (DSOS), and cuckoo optimization (CUK), with an SVM classifier for breast cancer detection using mRNA and micro-RNA expression data. The proposed algorithm selected 186 mRNAs out of 9,692 and 116 miRNAs out of 489 and obtained an accuracy above 90% for the miRNAs dataset and 100% for the mRNA dataset. Wei et al.^42^ proposed a generative adversarial model based on cancer genetic data (GANs). They used 12 different gene expression data from the TCGA, including lung, breast, prostate, colon, gastric, liver, rectal, esophageal, thyroid, clear cell renal cell carcinoma (CCRCC), uterine, and head and neck squamous cell carcinomas (HNSCC). They further used a reconstruction loss to enhance stability during model training. From their results, an accuracy of 92.6% was achieved by their proposed model. Deng et al.^43^ proposed a gene selection model in a two-stage format for cancer classification in microarray datasets. Their approach combined a multi-objective optimization genetic algorithm (XGBoost-MOGA) with gradient boosting (XGBoost). During the first stage, the XGBoost-based feature selection is used in ranking the genes to eliminate genes that are not relevant effectively, thereby leaving a group of genes that are most relevant to the class. In the second stage, a subset of optimal genes from the group of the most relevant genes is identified using XGBoost-MOGA through multi-objective optimization. Based on two widely used learning classifiers, a comparison of the proposed method with other state-of-the-art feature selection methods using two widely used learning classifiers on 14 publicly available microarray datasets was performed. The results demonstrated that XGBoost-MOGA outperformed previous methods in terms of accuracy, F-score, precision, and recall.

In Houssein et al.^44^, the selection of genes that contribute to the prediction of cancer from gene expression datasets with the highest accuracy based on microarray gene expression was achieved by combining a Barnacles Mating Optimizer (BMO) algorithm with SVM called (BMO-SVM). They evaluated the proposed model using four benchmark microarray datasets, including leukemia1, lymphoma, a small-round-blue-cell tumor (SRBCT), and leukemia2. From their results, the proposed BMO-SVM approach performed better than the other well-known methods, such as Particle Swarm Optimization (PSO), the Tunicate Swarm Algorithm (TSA), Artificial Bee Colony (ABC), and Genetic Algorithm (GA). Devi et al.^45^ proposed an Improved Whale Optimization Algorithm (IWOA) algorithm for gene selection. The proposed solution used a multi-objective fitness function that balances error rate minimization and feature selection. The results show that the proposed IWOA obtained a minimal subset of genes used for the BRCA classification using Gradient Boost Classifier (GBC) and achieved an accuracy of 97.7%. The related studies are summarised and presented in Table 1.

**Table 1:** Comparative summary of related existing studies.

Authors	Year	Method	Results	Limitation
Yuan et al.^19^	2020	SVM, RF	An accuracy of 100%	The algorithms used were not explicitly designed to address the unique challenges of gene expression data
Wang et al.^20^	2018	PDX		They didn't use the latest classifiers, and they used small samples
Danaee et al.^21^	2017	SDAE	An accuracy of 98.26%	The study does not explore using different optimization techniques or hyperparameter tuning to improve deep learning performance
Jia et al.^22^	2021	WGCNA	An accuracy of 97.36%	The study did not use hybrid classifiers based on deep learning, which is optimized using metaheuristic hyperparameter optimizers
Elbashir et al.^41^	2019	lightweight CNN	An accuracy of 98.76	The study does not explore using different optimization techniques or hyperparameter tuning to improve deep learning performance
Alshareef et al.^23^	2022	AIFSDL-PCD	An accuracy of 97.19%	Their study did not utilize a hybrid DL-based classifier with metaheuristics-based hyperparameter optimizers
MotieGhader et al.^33^	2020	PSO, ACO, ICA, WCC, LCA, GA, LA, FOA, DSOS, HTS, and CUK, with an SVM classifier	An accuracy of 90%	The study used a relatively small dataset
Wei et al.^42^	2022	GANs	An accuracy of 92.6%	The study did not use a combination of deep learning-based classifiers and metaheuristic-based hyperparameter optimizers
Deng et al.^43^	2022	XGBoost-MOGA	An accuracy of 56.67%	The number of samples used is less than 300
Houssein et al.^44^	2021	BMO-SVM	An accuracy of 99.36%	The study utilized small datasets, which may limit the generalizability of the findings
Devi et al.^45^	2023	IWOA	An accuracy of 97.7%	They evaluate their model on a small dataset (128 samples)

From the existing literature, various shortcomings were discovered regarding utilizing deep learning models for the given task. Deep learning models necessitate substantial data, and acquiring sizable, high-quality datasets for analyzing breast cancer gene expression can be challenging. Consequently, this can cause overfitting of the model to the training data, thereby resulting in inadequate performance on fresh, unobserved data. The computational complexity and time required for developing and training deep learning models can pose a significant hurdle to their widespread implementation in clinical practice. The complexity of breast cancer, which entails numerous biological processes such as cell proliferation, invasion, and angiogenesis, may not be captured entirely by deep learning models, thereby restricting their capacity to forecast outcomes or recognize potential therapeutic targets precisely. To resolve this challenge, optimizing the CNN model becomes necessary using suitable approximate optimization methods. Metaheuristic optimization algorithms have been applied to solve these problems. Nevertheless, the critical challenge of using deep learning models for effectively and efficiently classifying breast cancer remains unresolved. Therefore, this paper aims to enhance the efficacy of DL models on breast cancer detection and classification using gene expression data by leveraging a new optimization algorithm inspired by the biological mechanism of the Ebola disease.

## Model methodology

### Dataset and pre-processing 

Using the R software, we used the BRCA gene expression data from the Cancer Genome Atlas (TCGA) repository. The GDCquery function from the TCGAbiolinks library was used in developing the query^41,46^. The BRCA contains 1208 clinical samples and 14,895 genes or features. Moreover, there are 113 and 1095 normal and tumor samples, respectively. The data were identified to be noisy with many features. Therefore, different pre-processing steps were implemented to get clean data with genes positively contributing to BRCA detection. To identify the outliers samples, the array-array intensity correlation (AAIC), which defines a symmetric matrix of Spearman correlation between samples, was calculated^47^. The cut-off value of 0.6 was used to define the outlier samples to remove them. Normalization was applied for the gene expression data to ensure the validity of the expression levels and avoid biases in the analysis^48^. The TCGAanalyze-Normalization function was used from the TCGAbiolinks library to perform the normalization. Then filtration was performed using a cut-off value of 0.25 for reduction of gene number through the selection of genes whose mean expression values are higher than the cut-off value^41,49^. Consequently, the pre-processing obtained a dataset that contains 1208 clinical samples with 14,895 genes.

**Figure 1:**
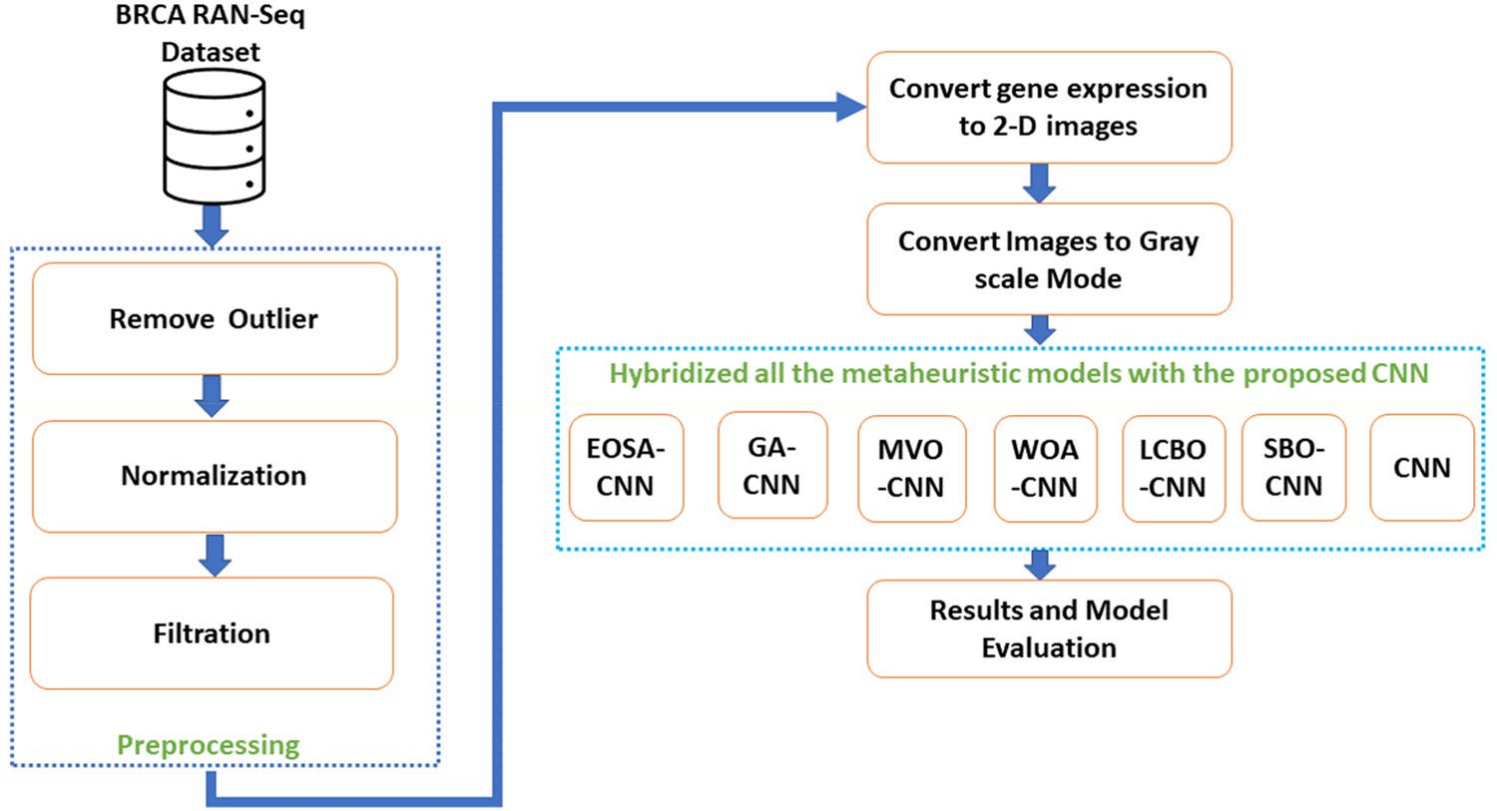
The proposed methodology.

The gene expression data was reshaped from 1 to 2D images with a dimension of 122 × 123 to be appropriate for our metaheuristic models. The BRCA gene expression data contains columns that could not be reshaped into the desired dimension. However, 112 columns of zeros were attached at the end to adjust the image size^41,50^. Moreover, we transformed the images into grayscale using the cvtColor() function from the OpenCV library in Python. This was done to ensure that the images met the requirements of the classification model and to improve image quality. Once the images were converted, they were prepared as input for the hybrid model. Figure 1 shows the proposed methodology.

### The CNN architecture

**Figure 2:**
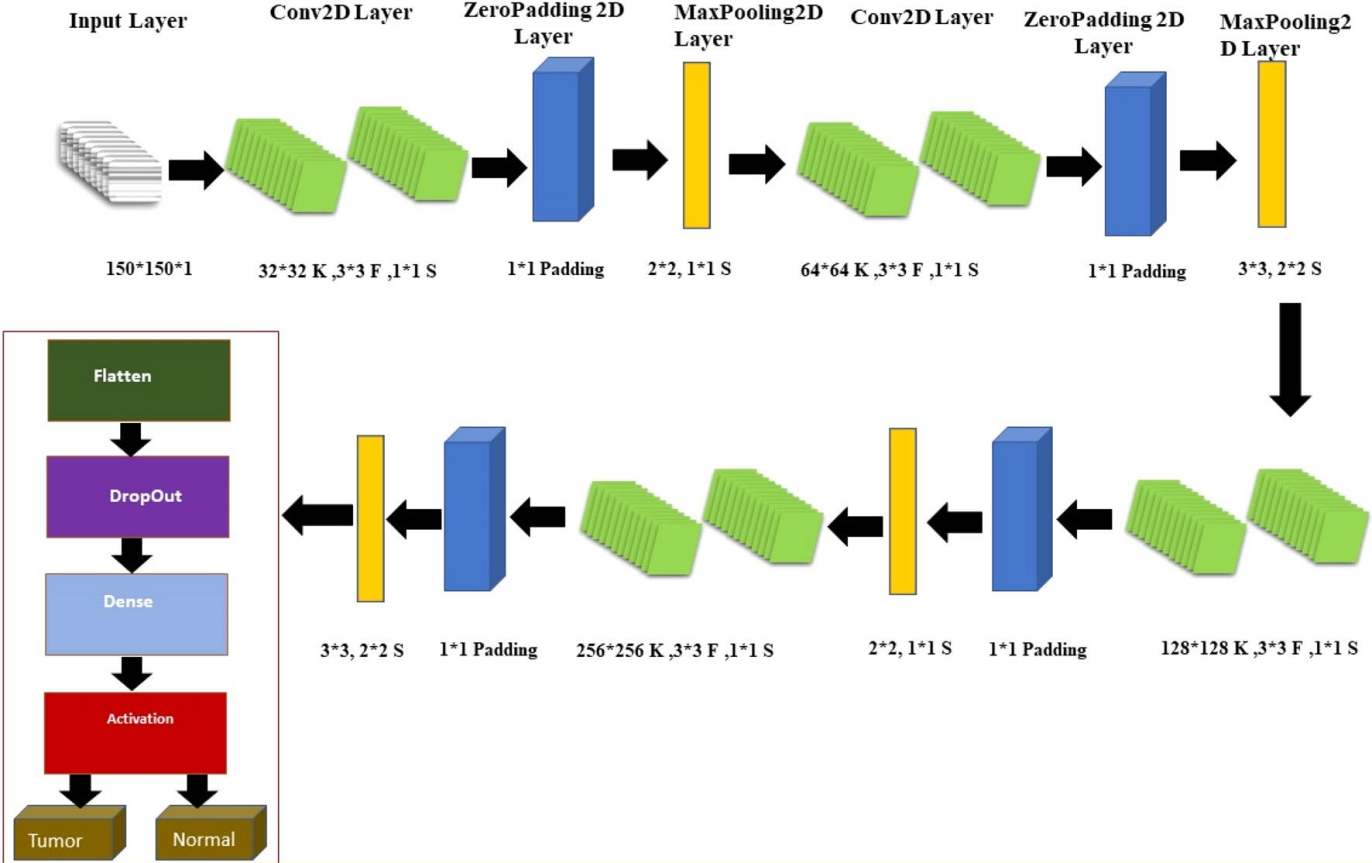
The proposed CNN architecture for the detection of breast cancer.

After the pre-processing step, the resulting images were used as input to the model. A specially designed CNN was used for the optimization model. The architecture of the proposed CNN model is a deep neural network designed to analyze and classify gene expression images with dimensions of $150 \times 150$ pixels and a single colour channel (grayscale). The model consists of multiple convolutional layers with increasing filter sizes, followed by max pooling layers to reduce the spatial dimensions of the feature maps. The architecture is designed to extract and learn high-level features from the input images, gradually increasing the number of filters to capture more complex patterns. The final output of the convolutional layers is flattened and passed through a Dropout layer, which randomly drops out some of the neurons to prevent overfitting. The final output layer is a Dense layer with ReLU activation that is fully connected. The CNN model architecture designed in this study is shown in Fig. 2. The proposed CNN model for breast cancer detection has a specific architecture that utilizes filters (denoted by "F"), kernels (denoted by "K"), and strides (denoted by "S").

### Ebola optimization search algorithm CNN model (EOSA-CNN)

Ebola is a viral hemorrhagic fever that affects humans and primates, also called Ebola hemorrhagic fever or Ebola virus disease. The Ebola viruses cause this disease, which can cause individuals to transition between susceptible, quarantined, infected, recovered, hospitalized, and deceased subpopulations in a seemingly random manner. Drawing inspiration from the Ebola virus's ability to spread effectively, a novel optimization algorithm that is both bio-inspired and population-based was developed. The method of the propagation of Ebola disease (EVD)^34^ was adopted in the design of the algorithm. To update the propagation, the EOSA model used a dynamic mechanism for propagation via susceptible, infection, quarantine, recovered, and hospitalized operations to gain a better fit. It helped to find the best or worst solution and provided an intuitive outcome. In this paper, the EOSA metaheuristic algorithm was hybridized with CNN to improve the performance of the CNN model. This was accomplished in all the iterations when the metaheuristic algorithm was trained to achieve the solution vector and update the CNN model. The weights and biases for the CNN were updated, and the loss function was subsequently calculated. Thereafter, the results obtained were compared with different hybrid models. The following steps describe the EOSA-CNN Model:

1. Set up the initial scalar and vector quantities for parameters and individuals, respectively. Assign initial values to individuals categorized as Susceptible (S), Infected (I), Recovered (R), Dead (D), Vaccinated (V), Hospitalized (H), and Quarantine (Q).
2. Randomly select an individual from the susceptible individuals as the index case (${\mathrm{I}}_{1}$)
3. Designate the index case as the global and current best, then compute its fitness value.
4. While there is at least one infected individual and the number of iterations is not complete,
  - Update the position of each susceptible individual based on their displacement, and generate newly infected individuals (nI) accordingly. Note that the greater the displacement of an infected case, the higher the infection rate, with shorter displacement representing exploitation and longer displacement signifying exploration.
    i. Based on (a), create individuals that are newly infected
    ii. The newly generated cases are then added to the newly infected individuals created in I.
  - Evaluate the number of individuals to be added to R, H, D, Q, V, and B determined by the size of I, based on their rates, respectively.
  - Use nI to update I and S.
  - Choose the current best from I and compare it with the global best.
  - While stopping criteria are not satisfied, return to step 4.
5. Return all solutions and the global best solution.

**Figure:**
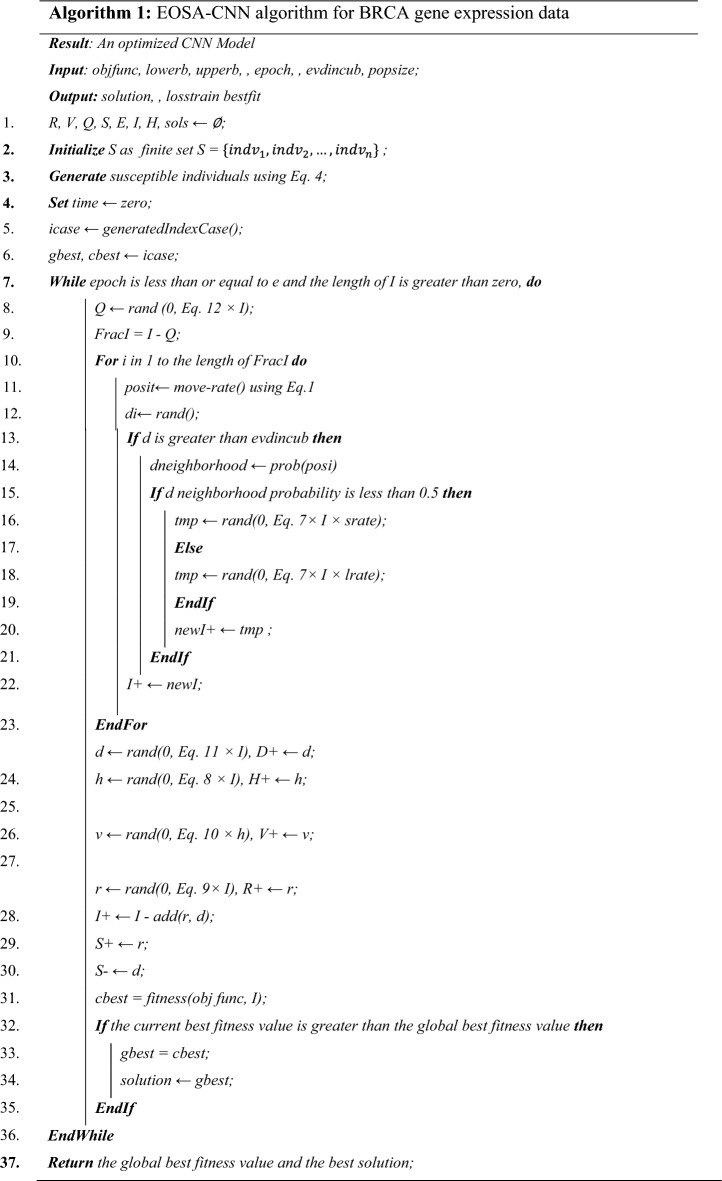

The pseudocode in Algorithm 1 presents the algorithm that uses mathematical models to optimize a CNN model. The algorithm uses evolutionary optimization techniques. The algorithm starts by initializing variables such as the CNN model's objective function, lower and upper bounds, batch size, number of epochs, population size, and the incubation period. It also creates empty sets for groups of individuals (Quarantine (Q), Susceptible (S), Exposed (E), Recovered (R), Hospitalized (H), Vaccinated(V), Infected (I)) and solutions. The set of susceptible individuals is then generated, and the algorithm starts with a time equal to 0 and an index case is randomly generated. The current best and global best solutions are set to the index case. The positions of the exposed individuals are updated by the algorithm using a mathematical model illustrated in Equation ${\mathrm{mI}}_{\mathrm{i}}^{\mathrm{t}+1}={\mathrm{mI}}_{\mathrm{i}}^{\mathrm{t}}+\mathrm{\rho M}$.

1 $$m{I}_{i}^{t+1}=m{I}_{i}^{t}+\rho M$$

The displacement scale factor of individuals is represented by $\rho$ while $m{I}_{i}^{t+1}$ and $m{I}_{i}^{t}$ indicate the updated and original positions at time $t$, respectively. The current time is denoted as $t+1$, and the movement rate of each individual represented as $M\left(I\right)$ is calculated using Eqs. (2) and (3).

2 $$M\left(I\right)=srate\times rand\left(\mathrm{0,1}\right)+M\left(In{d}_{best}\right)$$

3 $$M\left(S\right)=lrate\times rand\left(\mathrm{0,1}\right)+M\left(In{d}_{best}\right)$$

The exploration stage of the EOSA involves the infected individual moving beyond the normal neighbourhood range, $lrate$. In contrast, during the algorithm's exploitation phase, it is either assumed that the infected individual is displaced within a limit of $srate$ in comparison to its previous position and remains within a distance of zero (0).

4 $$individua{l}_{i}={L}_{i}+rand\left(\mathrm{0,1}\right)\times \left({U}_{i}+{L}_{i}\right)$$

The algorithm also uses Eq. (4) to generate the susceptible population, Eq. (5) computes the global best solution, and Eqs. (6), (7), (8), (9), (10), (11) and (12) update the population of the dead, infected, susceptible, hospitalized, recovered, vaccinated, quarantined, funeral, and exposed groups. These equations are scalar functions that represent each population's rate of change. Where ${U}_{i},{L}_{i}$ indicate the lower and upper for the ${i}^{th}$ individual, $i=\mathrm{1,2},..,\mathrm{N}.$

To determine the current best ($cBest$), the individuals infected in time t are evaluated, and the global best ($gBest$) is calculated using Eq. (5):

5 $$bestS=\left\{\begin{array}{c}gBest,\, If \,fitness(cBest) <fitness(gBest)\\ cBest,\, If \,fitness(cBest) \ge fitness(gBest)\end{array}\right.$$

At time t, the terms $cBest,$ $bestS$, and $gBest$ represent the current best solution, best solution, and global best solution, respectively. The objective function used for the problem is denoted by the term $fitness$.

The set of differential calculus equations used by the algorithm in updating the population of Quarantine (Q), susceptible (S), Infected (I), Recovered (R), Vaccinated (V), Dead (D), Funeral (F), Exposed (E), and Hospitalized (H) individuals as in Eqs. (6), (7), (8), (9), (10), (11) and (12).

6 $$\frac{\partial S\left(t\right)}{\partial t}=-\left({\beta }_{1}I+{\beta }_{3}D+{\beta }_{4}R+{\beta }_{2}\left(PE\right)\right)S-\left(\tau S+\Gamma I\right)$$

7 $$\frac{\partial I\left(t\right)}{\partial t}=\left({\beta }_{1}I+{\beta }_{3}D+{\beta }_{4}R+{\beta }_{2}\left(PE\right)\lambda \right)S-\left(\Gamma +\gamma \right)I-\left(\tau \right)S$$

8 $$\frac{\partial H\left(t\right)}{\partial t}=\alpha I-\left(\gamma +\varpi \right)H$$

9 $$\frac{\partial R\left(t\right)}{\partial t}=\gamma I-\Gamma R$$

10 $$\frac{\partial V\left(t\right)}{\partial t}=\gamma I-\left(\mu +\vartheta \right)V$$

11 $$\frac{\partial D\left(t\right)}{\partial t}=\left(\tau S+\Gamma I\right)-\delta D$$

12 $$\frac{\partial Q\left(t\right)}{\partial t}=\left(I-\left(\gamma R+\Gamma D\right)\right)-\xi Q$$

Equations (6), (7), (8), (9), (10), (11) and (12) $\frac{\partial \mathrm{I}\left(\mathrm{t}\right)}{\partial \mathrm{t}}=\left({\upbeta }_{1}\mathrm{I}+{\upbeta }_{3}\mathrm{D}+{\upbeta }_{4}\mathrm{R}+{\upbeta }_{2}\left(\mathrm{PE}\right)\uplambda \right)\mathrm{S}-\left(\Gamma +\upgamma \right)\mathrm{I}-\left(\uptau \right)\mathrm{S}$ are scalar functions. For each function, a single float value is assigned. The rate at which the susceptible population changes is specified, and it is used to determine the number of susceptible individuals at time t by applying it to the susceptible vector's current size. The sets of individuals in vectors I, H, R, V, D, and Q is calculated using this procedure. It is assumed that the initial conditions of $S\left(0\right)=S0,I\left(0\right)=I0,R\left(0\right)=R0,D\left(0\right)=D0,P\left(0\right)=P0,andQ\left(0\right)=Q0$, where $t$ follows after the epoch, and the term $\delta$ in Eq. (11) represents the burial rate. The quarantine rate for infected Ebola cases is denoted by Eq. (12).

## Experimentation, results and discussion

### System configuration and algorithms parameters setting

The experiments were conducted using Dell Optiplex 5050 computer machine with the following configuration: an Intel Core i5 7th generation processor with a hard disk size of 500 GB and 16 GB memory. All the models were developed using Python. EOSA-CNN model's performance was compared to that of a standalone CNN and five other metaheuristic algorithms, namely MVO-CNN (Physics-based), GA-CNN (Evolutionary-based), LCBO-CNN (Human-based), WOA-CNN (Swarm-based), and SBO-CNN (Biology-based. The same parameter values of batch size and epoch were used for all algorithms. The input images to the hybrid algorithms were of size 150 × 150, corresponding to the pre-processed images. The configuration of metaheuristic algorithms and the EOSA-CNN algorithm for optimizing the proposed CNN model is depicted in Table 2 below. Table 3 presents the CNN hyperparameter configuration.

**Table 2:** Parameters configuration of the hybrid algorithms.

Parameter	Value
EOSA-CNN algorithm	
Epxilon	0.001
Batch size	128
Epoch	100
GA-CNN algorithm	
Crossover percentage on the population	0.95
Mutation percentage	0.025
Lower and upper domain ranges	[(1, 1)]
Batch size	128
Epoch	100
WOA-CNN algorithm	
A	Decreased linearly from 2 to 0
Rang of lower and upper domain	[(1, 1)]
Batch size	128
Epoch	100
SBO-CNN algorithm	
Alpha	[0.94]
Z	[0.02]
Mutation probability	[0.05]
Lower and upper domain ranges	[(1, 1)]
Batch size	128
Epoch	100
MVO-CNN algorithm	
Wep min–max	(1.0, 0.2)
Lower and upper domain ranges	[(1, 1)]
Batch size	128
Epoch	100
LCBO-CNN algorithm	
r1	2.35
Batch size	128
Epoch	100

**Table 3:** The proposed CNN hyperparameter configuration.

Hyperparameter	Value
Learning rate	0.001
Epoch	5
Batch size	32
Optimizer	Adam
Kernel size	[3, 3]
Convolution layers	[2conv-2conv]
Activation function	Relu
Pooling layers	[(2,2), (3,3)]
Padding/Stride	Same/(1,1)

### Model performance measuring metrics

To evaluate the efficacy of the model, Balanced Accuracy, Accuracy, precision, Recall, f1-score, Cohen's kappa, sensitivity, and specificity are calculated. The false positive (FP) indicates the number of images incorrectly predicted as cancerous when they are not, while the true positive (TP) denotes the number of accurately classified cancerous images. False negative (FN) represents the number of cancerous images that were misclassified as non-cancerous. True negative (TN) is the number of accurately classified non-cancerous images. The performance metrics are calculated using the formulas involving TP, FP, FN, and TN presented in Eqs. (13), (14), (15), (16), (17), (18), (19) and (20).

13 $$Accuracy=\frac{TP+TN}{TP+TN+FP+FN}$$

14 $$Kappa=\frac{{\text{Accuracy}}-\text{Random Accuracy}}{1-\text{Random Accuracy}}$$

15 $$Random\,Accuracy=\frac{{\text{ActNegative}}\times {\text{PredNegave}}+{\text{PredPositive}}\times {\text{ActPositive}}}{{\text{Total}}\times {\text{Total}}}=\frac{\left(FP+TN\right)\times \left(FN+TN\right)+\left(TP+FN\right)\times \left(TP+FP\right)}{\left(TP+TN+FP+FN\right)\times \left(TP+TN+FP+FN\right)}$$

16 $$Specificity=\frac{TN}{\left(TN+FP\right)}$$

17 $$Sensitivity=Recall=\frac{TP}{\left(TP+FN\right)}$$

18 $$Precision=\frac{TP}{TP+FP}$$

19 $$F1Score=\frac{2*\left(Recall*Precision\right)}{\left(Recall+Precision\right)}$$

20 $$Balanced\,Accuracy=\frac{Sensitivity+Specificity}{2}$$

## Results and discussions

Table 4 presents the overall performance of the competing algorithms. It shows that the hybrid algorithms performed better than the traditional CNN and the proposed model EOSA-CNN recorded a better performance than the hybrid algorithms. We calculate the Balanced Accuracy, Accuracy, precision, Recall, f1-score, Cohen's kappa, sensitivity, and specificity. In terms of Balanced Accuracy, WOA-CNN, GA-CNN, MVO-CNN, SBO-CNN, CNN, and LCBO-CNN achieved 0.956, 0.942, 0.923, 0.942, 0.924, 0.940, respectively. Whereas the EOSA-CNN achieved 0.958, which is the best performance. With reference to accuracy, the GA-CNN, SBO-CNN, and EOSA-CNN performed the same result of 0.983. In contrast, for recall, EOSA-CNN and WOA-CNN attained 0.928. In terms of the f1-score, EOSA-CNN achieved 0.912.

**Table 4:** The overall performance of the algorithms.

Methods	MVO-CNN	GA-CNN	SBO-CNN	WOA-CNN	CNN	LCBO-CNN	EOSA-CNN
Balanced accuracy	0.923	0.943	0.943	0.957	0.925	0.941	0.959
Accuracy	0.977	0.983	0.983	0.980	0.980	0.980	0.983
Precision	0.889	0.926	0.926	0.867	0.923	0.893	0.897
Recall	0.857	0.893	0.893	0.929	0.857	0.893	0.929
f1-scores	0.873	0.909	0.909	0.897	0.889	0.893	0.912
Cohens kappa	0.860	0.900	0.900	0.886	0.878	0.882	0.903
Sensitivity	0.989	0.993	0.993	0.985	0.993	0.989	0.989
Specificity	0.857	0.893	0.893	0.929	0.857	0.893	0.929

**Figure 3:**
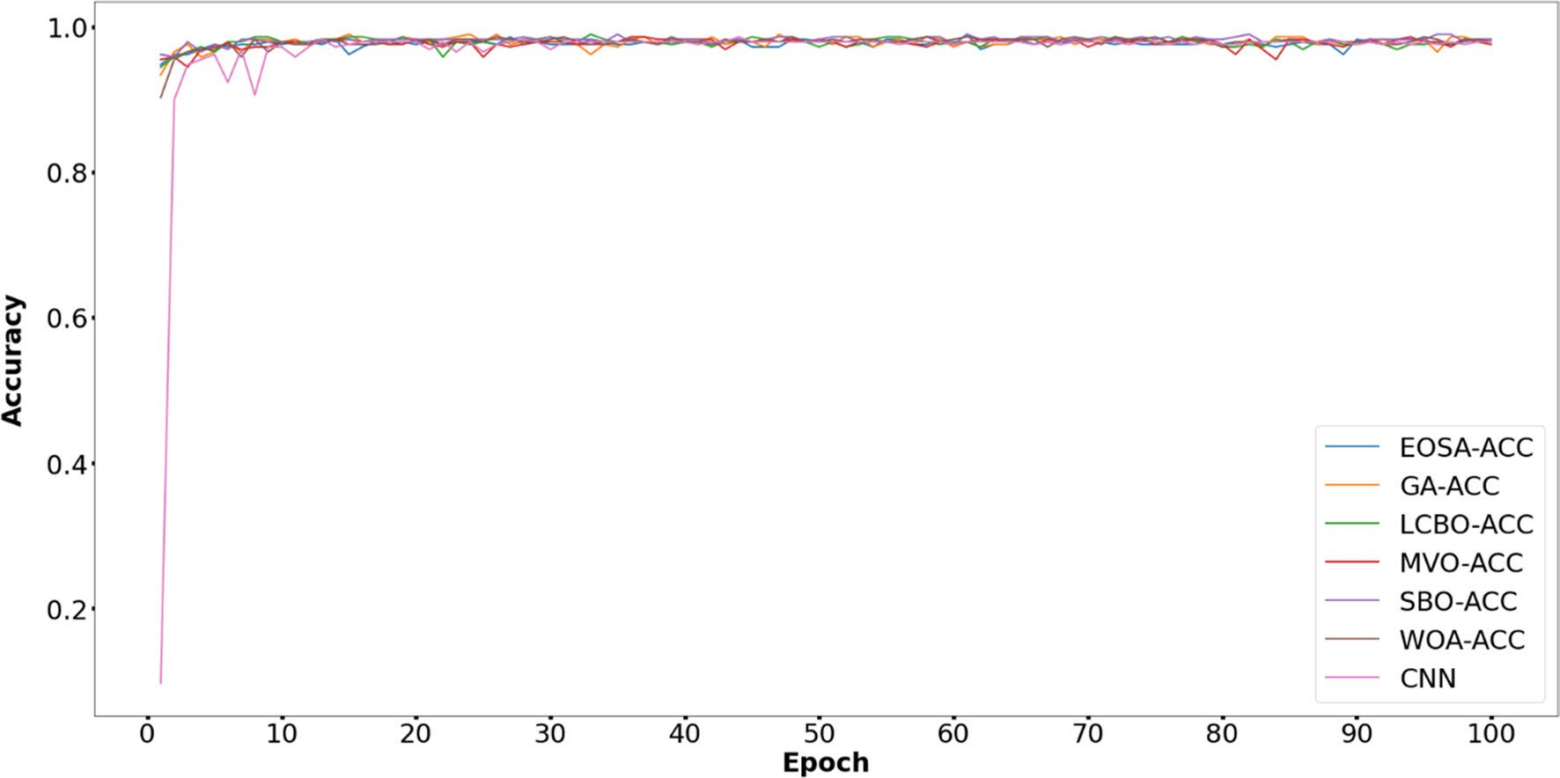
Comparative performance of the proposed EOSA-CNN model against other models.

The comparative study of the proposed method with five metaheuristic algorithms and CNN is reported in Fig. 3. The proposed model performs better than the other models with respect to the validation accuracy in 100 epochs.

**Figure 4:**
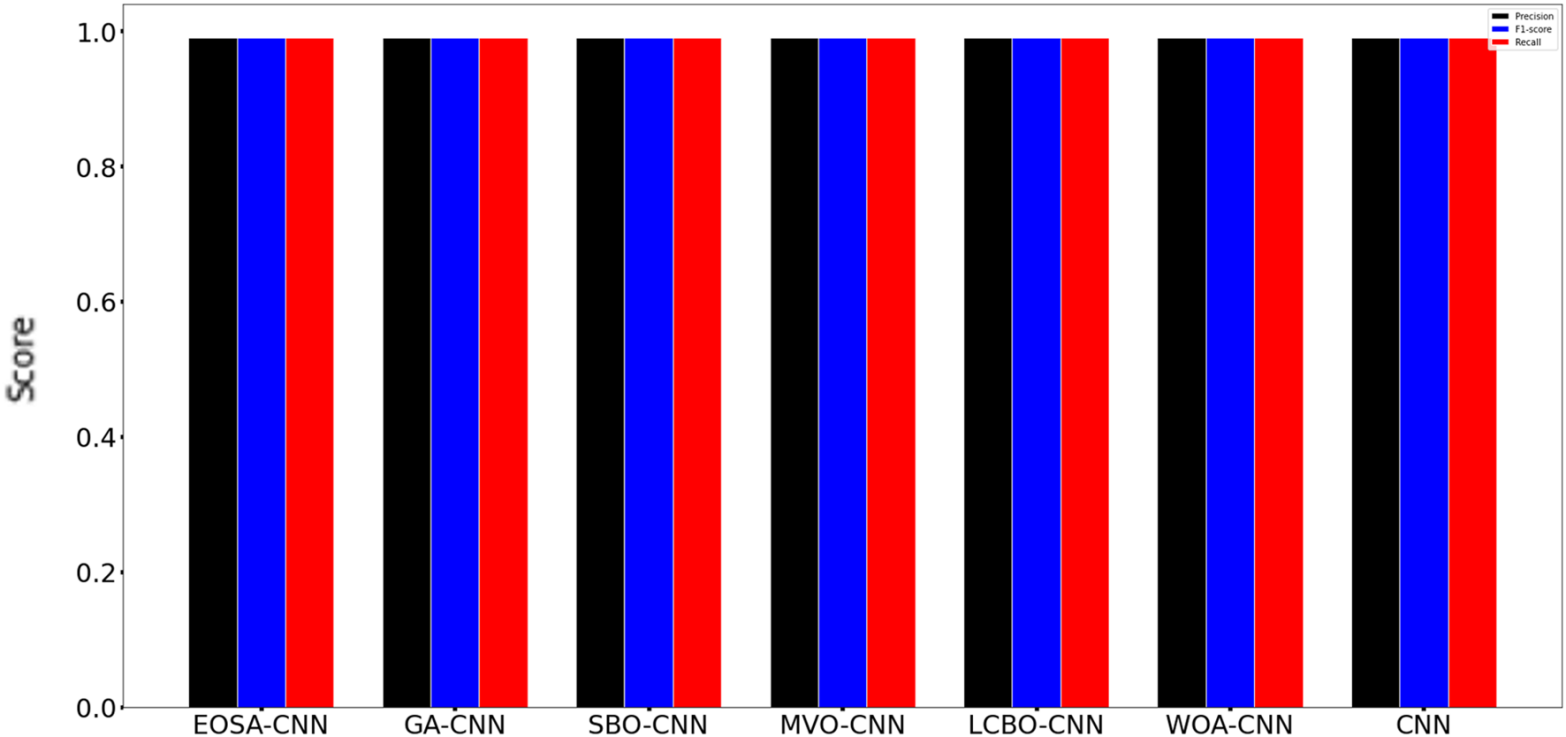
Comparative results of precision, f1-score, and recall for EOSA-CNN model and other models for normal class.

Figure 4 presents the precision, f1-score and recall of all models per normal class. It shows that the Precision of the GA-CNN and SBO-CNN have the same performance of 0.93 and CNN performance of 0.92. Furthermore, the gene expression dataset was imbalanced, so different metrics were calculated for more confirmation, like F1-Score, balanced accuracy, and recall. It presents the F1-score result of EOSA-CNN has a high performance of 0.91 for the normal class. Also, GA-CNN and SBO-CNN have identical results. The EOSA-CNN have high performance compared to other methods in term of Recall 0.93%. All the methods correctly identified the tumor class with a high performance of 99% in terms of recall, precision, and F1-Score. Overall, the experiments indicated that the hybrid models benefited from pre-processing the gene expression data and almost had an equivalent performance in detecting the BRCA.

**Figure 5:**
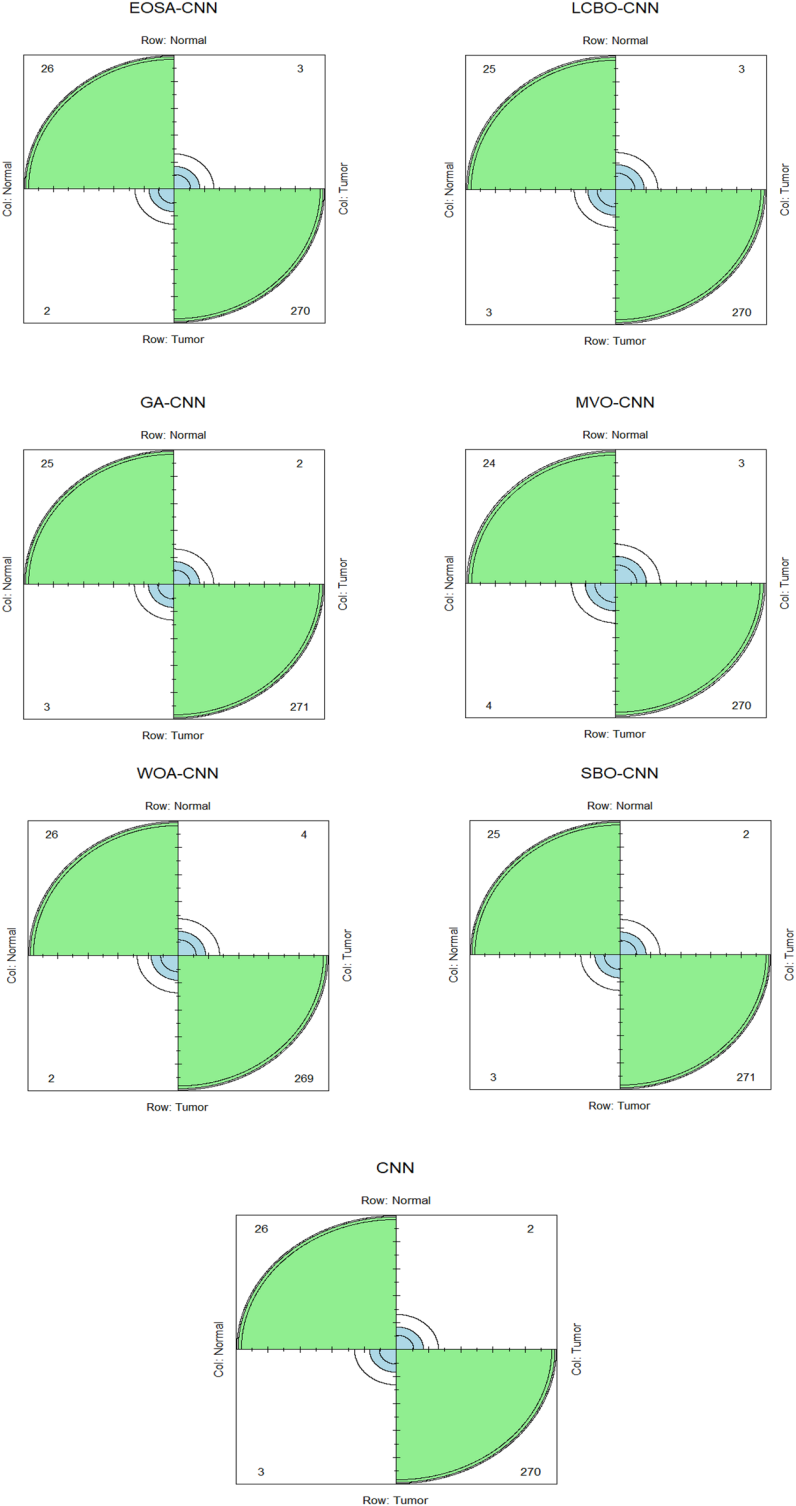
Confusion matrix (Hybrid algorithms and CNN).

Figure 5 shows the confusion matrix for CNN and the hybrid algorithm, considering all the datasets' class labels. Each plot of the confusion matrix shows the classification accuracy for all classes, providing an accurate performance report for each one. Taking EOSA-CNN (top left of Fig. 5), for instance, the hybrid algorithm proposed in this study correctly identified 26 from 28 samples as a normal class and 270 from 273 samples as tumor. Also, CNN correctly identified the tumor class but misclassified 3 from 29 samples for the normal class. This result highlights the significance of the proposed hybrid algorithm in this study as it successfully enhanced the classification accuracy.

**Figure 6:**
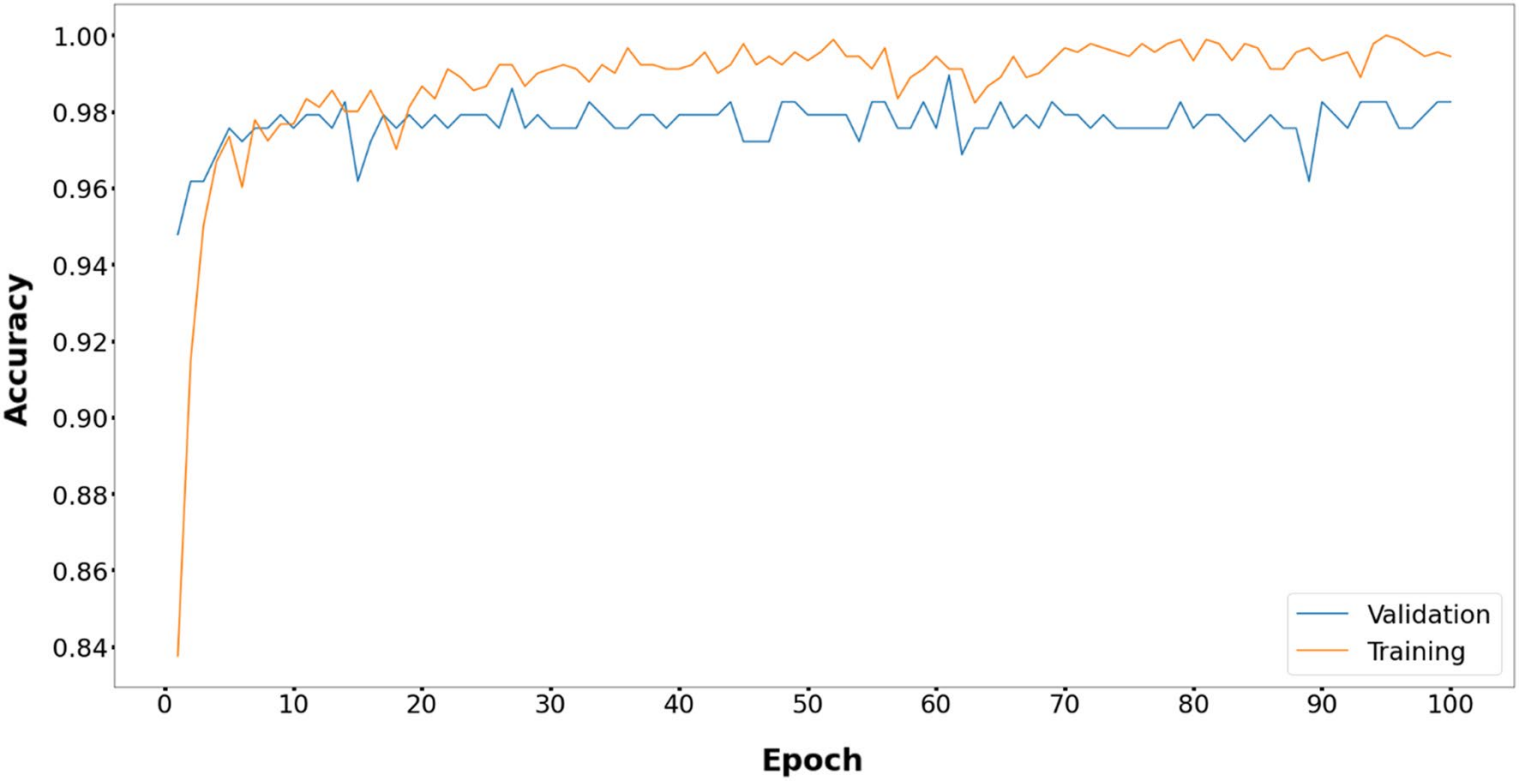
Training and validation accuracy curve for EOSA-CNN.

**Figure 7:**
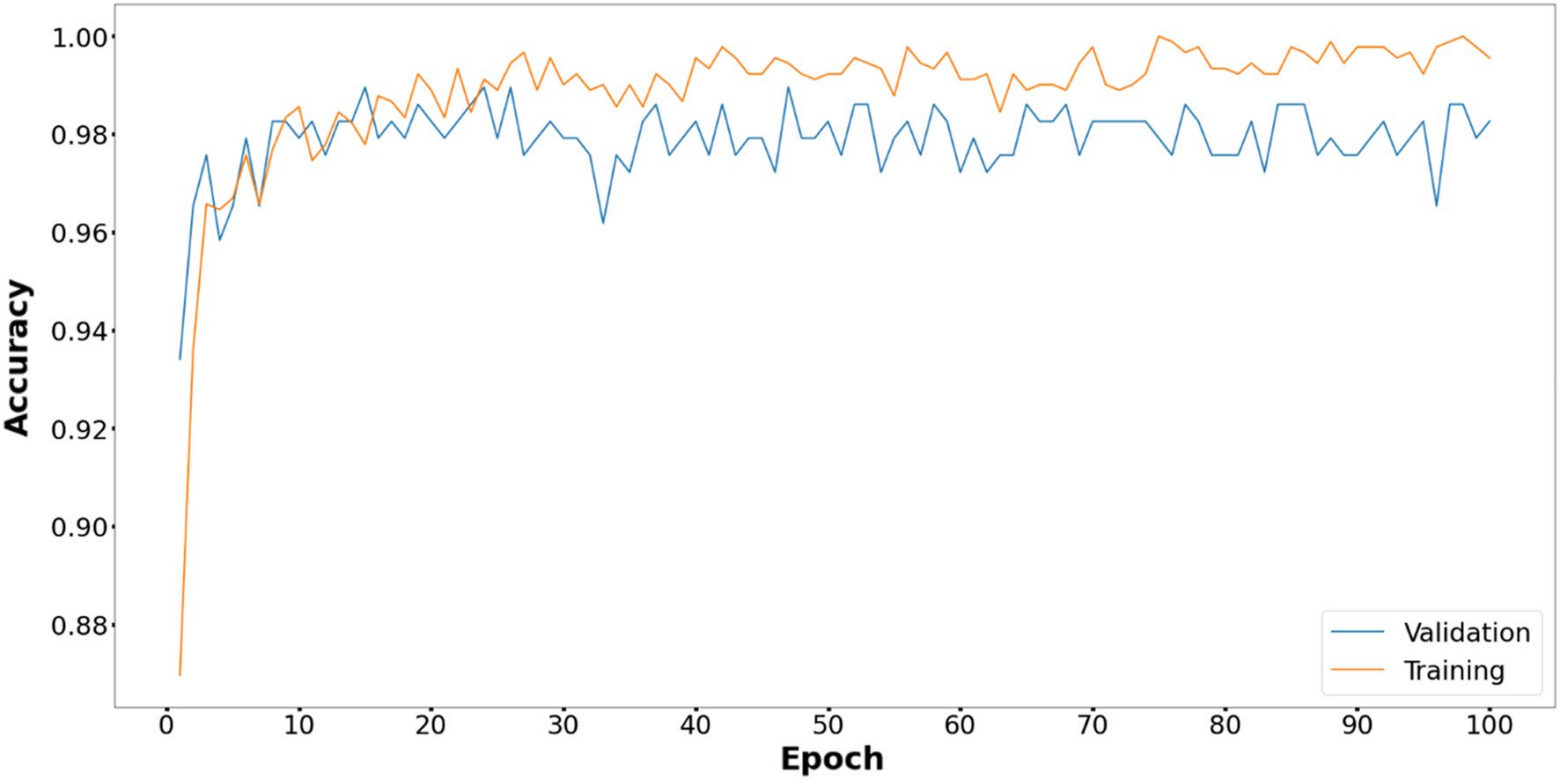
Training and validation accuracy curve for GA-CNN.

**Figure 8:**
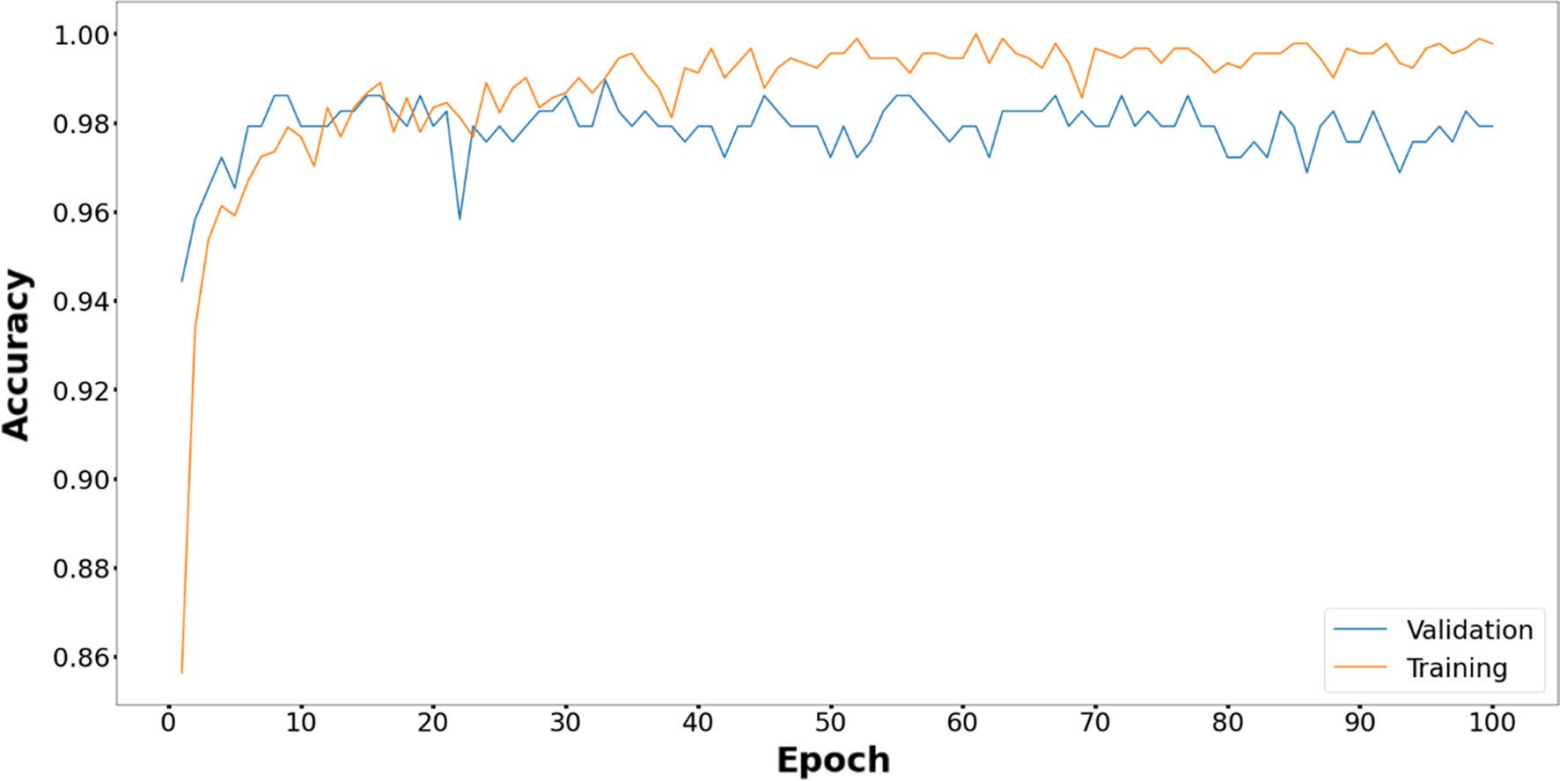
Training and validation accuracy curve for LCBO-CNN.

**Figure 9:**
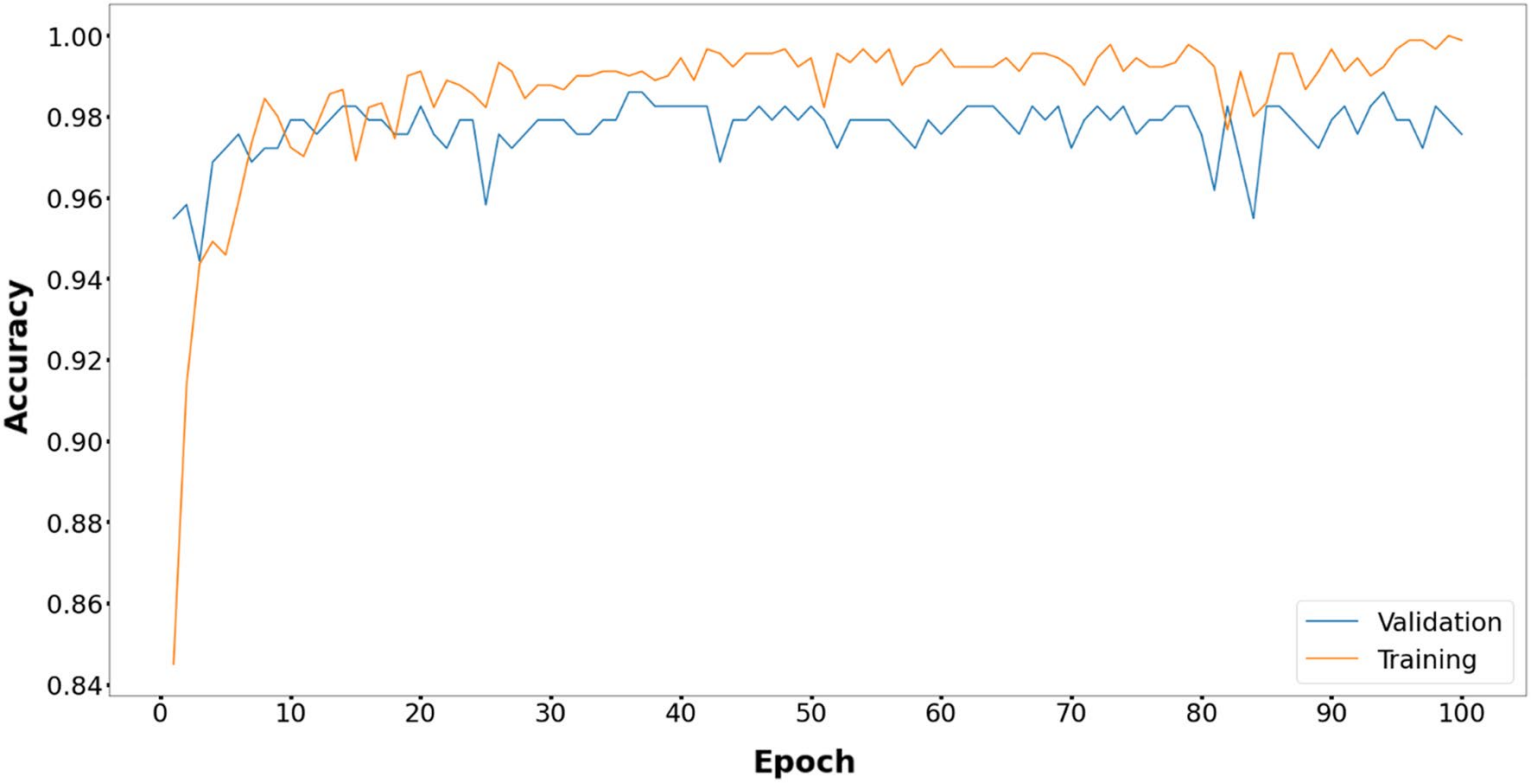
Training and validation accuracy Curve for MVO-CNN.

**Figure 10:**
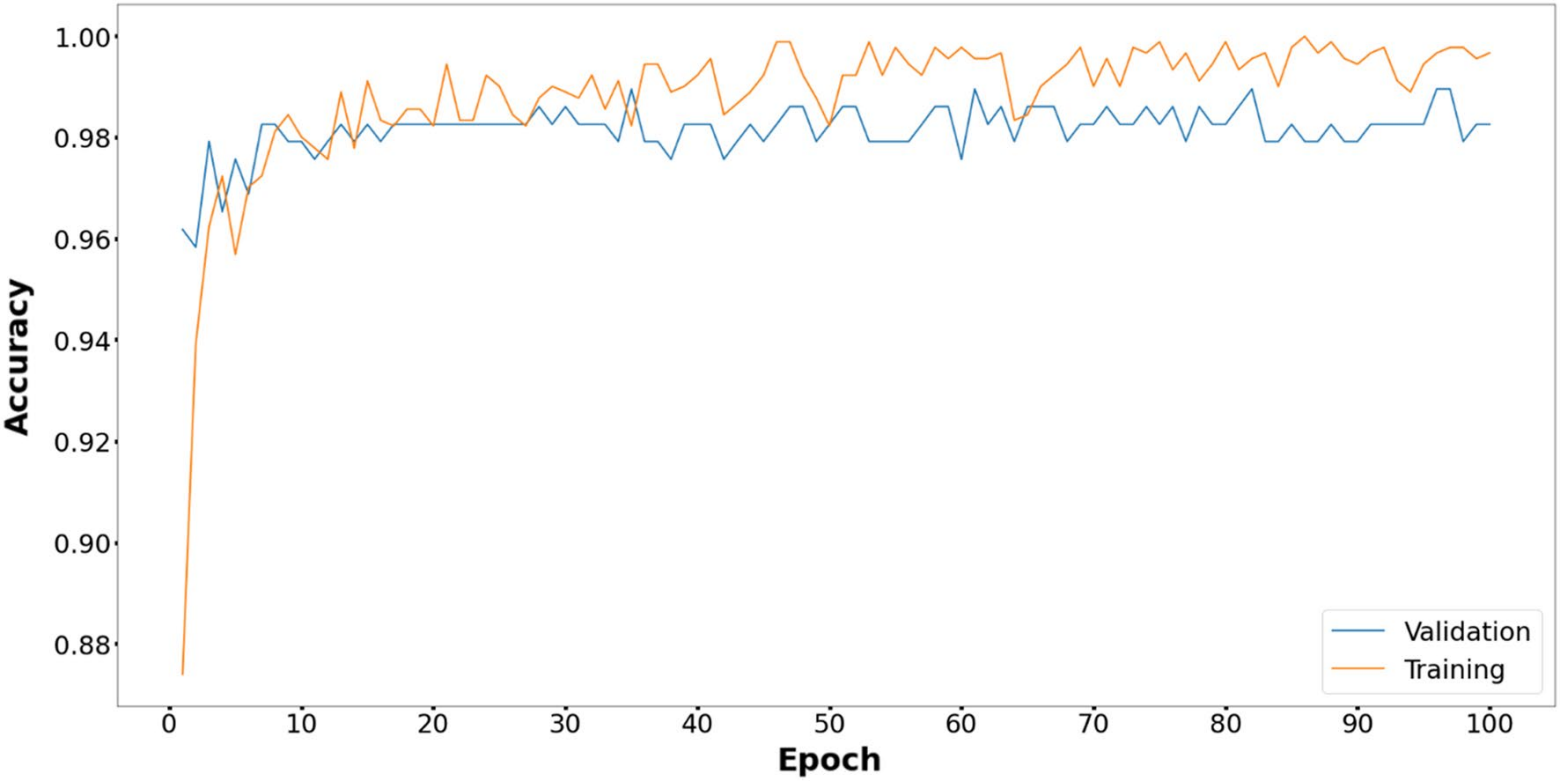
Training and validation accuracy curve for SBO-CNN.

**Figure 11:**
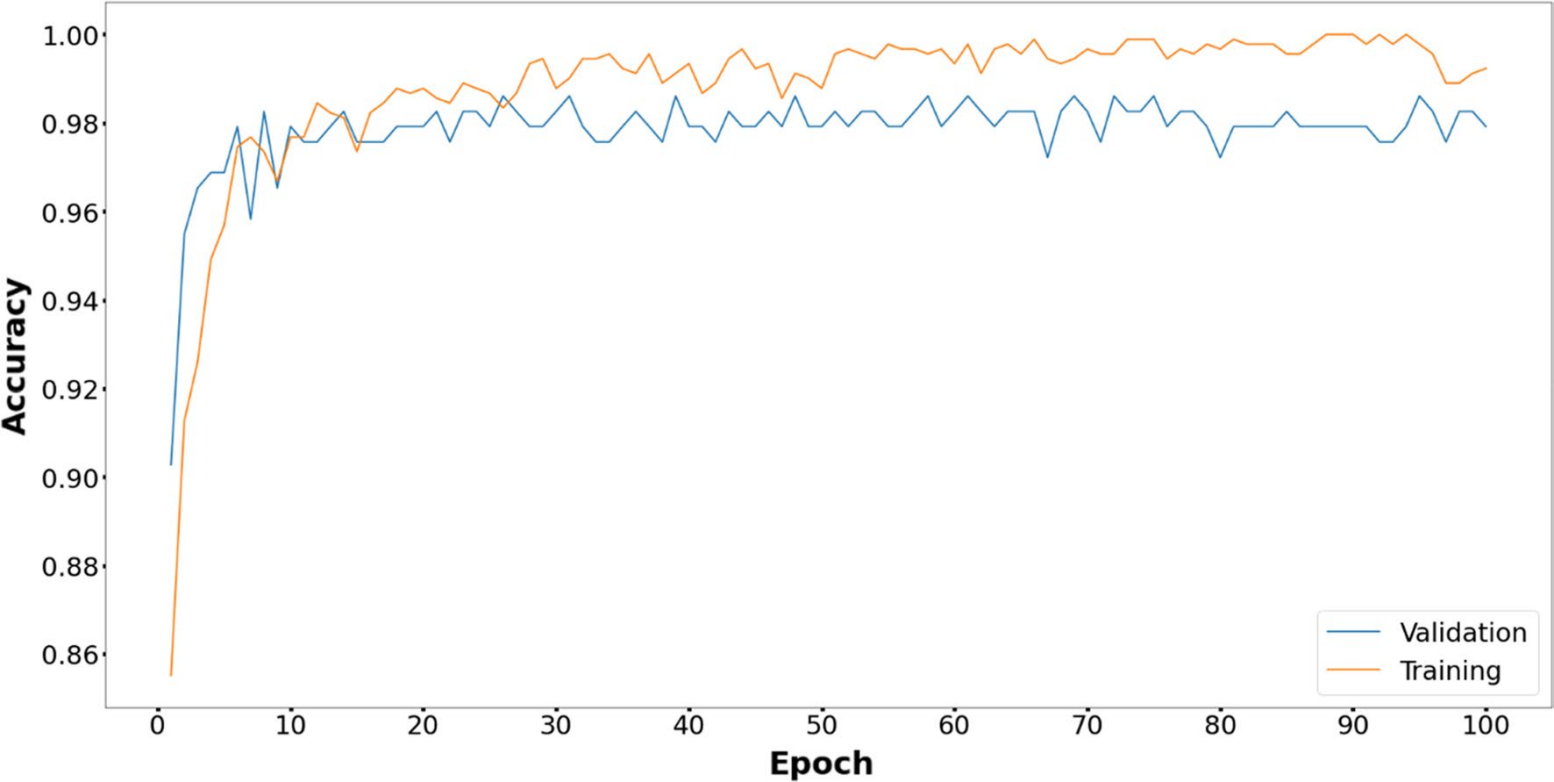
Training and validation accuracy curve for WOA-CNN.

**Figure 12:**
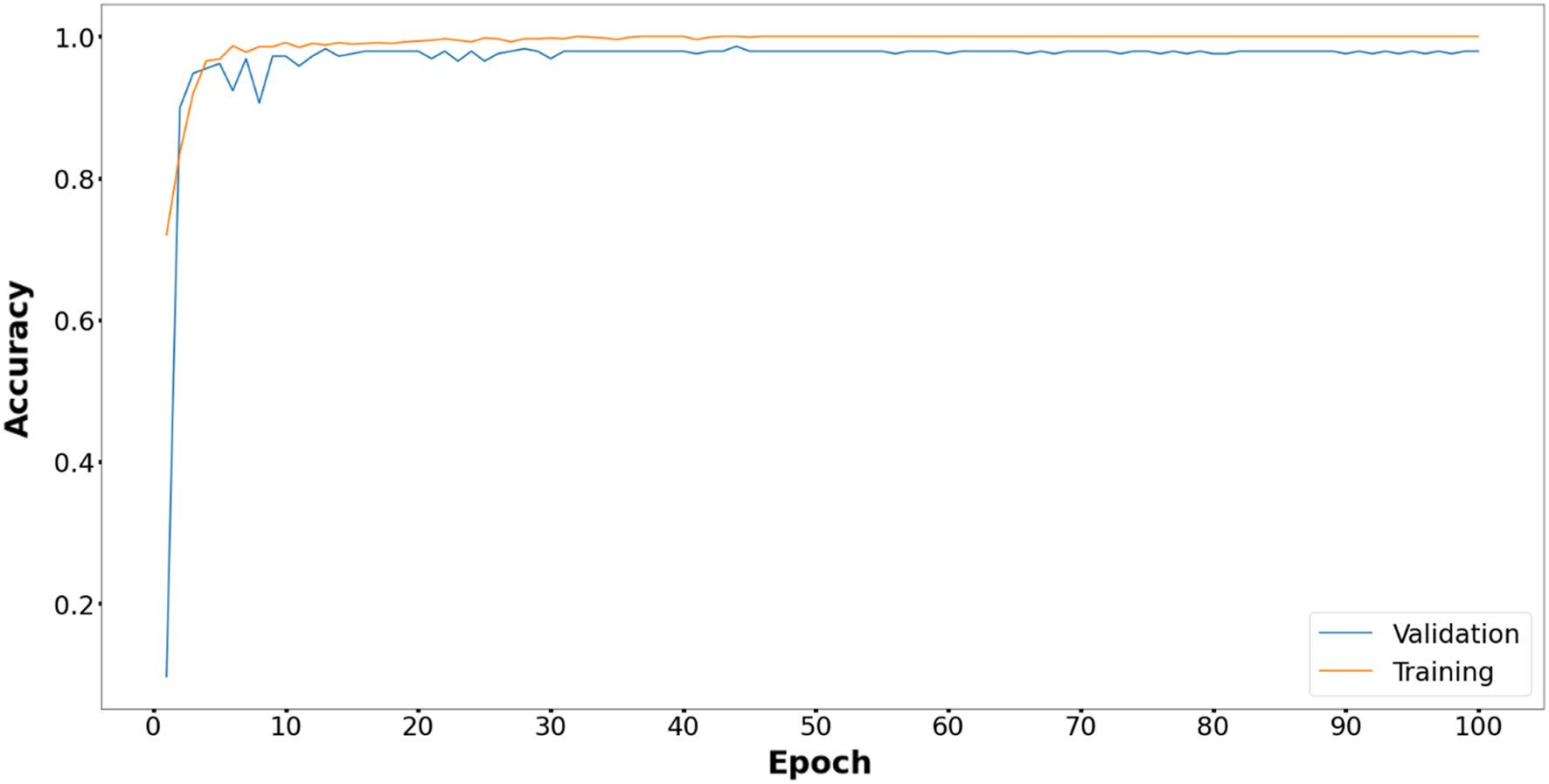
Training and validation accuracy curve for CNN.

Figures 6, 7, 8, 9, 10 and 11 display the training and validation accuracy for all hybrid algorithms in each epoch. In all the hybrid models, the validation accuracy is higher than the training accuracy at the beginning of training. That indicates the models possess good generalization ability to new, unseen data, which is a positive indication. During training, the model's training accuracy improves, while the validation accuracy improves slower. Both training and validation accuracies stabilize at a level higher than 97%. In Fig. 12, CNN's performance in training and validation is depicted. Although the training accuracy improves and reaches 100%, the validation accuracy remains lower. This implies that the model is overfitting to the training data, effectively memorizing it but lacking the ability to perform well on new and unseen data. As a result, it may lack generalization ability.

### Comparison with related studies

Table 5 shows the comparison between our proposed model performance and different studies. The proposed model in this study achieved higher classification accuracy than the results observed in previous works reported by Danaee et al.^21^, Jia et al.^22^, and MotieGhader et al.^33^. While Elbashir et al.^41^ achieved higher classification accuracy than our study using a CNN model, our approach showed a sensitivity of 0.9890% and an f1-score of 0.99% for both tumor and normal class. Moreover, the EOSA-CNN model achieved a sensitivity of 0.989%, which means the model has missed a few of the positive cases. Sensitivity is a crucial metric as it assesses the model's ability to detect positive cases correctly. Our models must identify all positive cases to ensure accurate predictions. Thus, this study highlights the significance of employing a metaheuristic algorithm to optimize CNN model hyperparameters, which is crucial in selecting the optimal combination of biases and weights required to train a CNN model effectively. Furthermore, the proposed method showcased that integrating these methods can significantly enhance gene expression data's overall performance and classification accuracy.

**Table 5:** A comparison of our model performance with several models used for gene expression data classification.

Author	Dataset	Methods	Result
Danaee et al.^21^	TCGA BRCA	SDAE method with SVM-RBF	Accuracy of 98.26%
Jia et al.^22^	TCGA BRCA	SVM, DT, BN, ANN, CNN-leNet and CNN-alexNet	Average accuracy of 97.36%
MotieGhader et al.^33^	mRNA and micro-RNA expression data	(WCC, LCA, GA, PSO, ACO, ICA, LA, HTS, FOA, DSOS, and CUK) with an SVM classifier	All algorithms achieved accuracy above 90% for the miRNA
Elbashir et al.^41^	TCGA BRCA	Lightweight CNN model	Accuracy of 98.76%, Sensitivity of 91.43%, and F-measure of 95.5%
Proposed method	TCGA BRCA	EOSA-CNN	Accuracy of 98.3%, precision of 99%, f1-score of 99%, kappa of 90.3%, specificity of 92.8%, recall of 99%, and sensitivity of 98.9%

### Strength and limitations of the EOSA-CNN model

In this section, the limitations of the study are discussed in more detail, including the small sample size of gene expression data compared to the very high number of genes. Moreover, the absence of addressing the problem of imbalanced data using approaches such as random over and under-sampling and cluster-based over-sampling is considered a serious challenge. The sample size used for the study may not be sufficient to capture the full complexity of the gene expression data, leading to potential biases and limitations in the analysis. Additionally, the issue of imbalanced data can significantly impact the model's performance, as the algorithm may be biased towards the majority class and struggle to predict the minority class accurately. While the EOSA-CNN model outperformed traditional CNN models and other hybrid algorithms, there is still room for improvement in addressing these limitations. Future research should concentrate on more experiments using large sample sizes of genomics data with handling class imbalance to enhance the model's effectiveness. Despite this constraint, the EOSA-CNN model outperformed other hybrid algorithms and traditional CNN models. Furthermore, evaluating the EOSA algorithm's performance in diverse diseases and medical conditions would be crucial to assess its generalizability and applicability to a broader range of healthcare problems. By addressing these limitations and exploring the model's performance in various contexts, the EOSA-CNN model could be a promising tool for accurate and reliable disease diagnosis and classification based on gene expression data.

## Conclusion and future work

Breast cancer is the most common medical diagnosis in women. The study, understanding and research of breast cancer have aided the diagnosis and development of new treatments for breast cancer. Gene expression profiling is helping researchers and doctors to comprehend the heterogeneous nature of breast cancer on a genomic level. In this study, we developed a hybrid model that combines the Ebola optimization search algorithm (EOSA) with CNN architecture for the detection of breast cancer and diagnosis using gene expression data. We prepared the data using different pre-processing methods, including removing the outliers using Array-Array Intensity Correlation (AAIC). To avoid biases in the expression measures, we utilized the normalization method. The final step in pre-processing was filtration. After that, we converted the gene expression data into two-dimensional images, which were converted into grayscale images. For the classification, we use the EOSA-CNN model. The findings of this study demonstrate that the proposed model achieved high-performance measurements with exceptional accuracy (98.3%), precision (99%), recall (99%), f1-score (99%), kappa (90.3%), specificity (92.8%), and sensitivity (98.9%) for the cancerous class. These results suggest that the model has the potential to be an effective and reliable method for breast cancer detection using gene expression data. For future extensions, we planned to solve the problem of imbalanced data and hybridize the model with various state-of-the-art optimization algorithms.

## Data Availability

The dataset is publicly available on The Cancer Genome Atlas (TCGA) repository.

## Abbreviations

CNN: Convolutional neural network
TCGA: The cancer genome atlas
AAIC: Array-array intensity correlation
EOSA: Ebola optimization search algorithm
GA: Genetic algorithm
LCBO: Life choice-based optimization
MVO: Multi-verse optimizer
SBO: Satin bowerbird optimization
WOA: Whale optimization algorithm
BRCA: Breast cancer
WHO: World health organization
NGS: Next generation sequencing
RNA-Seq: Ribonucleic acid sequencing
MCFS: Monte Carlo feature selection
RF: Random forest
SVM: Support vector machine
SDAE: Stacked denoising autoencoder
GEO: Gene expression omnibus
WGCNA: Weighted gene co-expression network analysis
AIFSDL-PCD: Intelligence-based feature selection method with a deep learning model for prostate cancer detection
NP-hard: Nondeterministic polynomial time hard
WCC: World competitive contest
LCA: League championship algorithm
PSO: Particle swarm optimization
ACO: Ant colony optimization
ICA: Imperialist competitive algorithm
LA: Learning automata
HTS: Heat transfer optimization algorithm
FOA: Forest optimization algorithm
DSOS: Discrete symbiotic organisms search
CUK: Cuckoo optimization
EVD: Ebola virus disease
PDX: Patient-derived tumor xenograft
ANN: Artificial neural network
PPI: Protein–protein interaction
DT: Decision tree
BN: Bayesian network
CIWO: Chaotic invasive weed optimization
FS: Feature selection
NNNSGAII: Non-dominated sorting genetic algorithm—II
GANs: Generative adversarial model based on cancer genetic data
CCRCC: Clear cell renal cell carcinoma
HNSCC: Head and neck squamous cell carcinomas
XGBoost: Gradient boosting
MOGA: Multi-objective optimization genetic algorithm
BMO: Barnacles mating optimizer
SRBCT: Small-round-blue-cell tumor
TSA: Tunicate swarm algorithm
ABC: Artificial bee colony
IWOA: Improved whale optimization algorithm
GBC: Gradient boost classifier
1D: One-dimensional
2D: Two-dimensional
nI: Infected individuals
TP: True positive
FP: False positive
FN: False negative
TN: True negative
